# Case report: Artificial thymic organoids facilitate clinical decisions for a patient with a *TP63* variant and severe persistent T cell lymphopenia

**DOI:** 10.3389/fimmu.2024.1438383

**Published:** 2024-09-18

**Authors:** Alevtina Gall, Marita Bosticardo, Stacey Ma, Karin Chen, Kayla Amini, Francesca Pala, Ottavia M. Delmonte, Tara Wenger, Michael Bamshad, John Sleasman, Matthew Blessing, Nicolai S. C. van Oers, Luigi D. Notarangelo, M. Teresa de la Morena

**Affiliations:** ^1^ Division of Immunology, Department of Pediatrics, University of Washington and Seattle Children’s Hospital, Seattle, WA, United States; ^2^ Laboratory of Clinical Immunology and Microbiology, Division of Intramural Research, National Institute of Allergy and Infectious Diseases, National Institutes of Health (NIH), Bethesda, MD, United States; ^3^ Division of Allergy and Infectious Diseases, Department of Internal Medicine, University of Washington, Seattle, WA, United States; ^4^ Division of Genetic Medicine, Department of Pediatrics, University of Washington and Seattle Children’s Hospital, Seattle, WA, United States; ^5^ Brotman Baty Institute for Precision Medicine, University of Washington, Seattle, WA, United States; ^6^ Department of Pediatrics, Duke University Medical Center, Durham, NC, United States; ^7^ Division of Craniofacial Medicine, Department of Pediatrics, University of Washington and Seattle Children’s Hospital, Seattle, WA, United States; ^8^ Department of Immunology, Pediatrics and Microbiology, University of Texas Southwestern Medical Center, Dallas, TX, United States

**Keywords:** *TP63*, T cell lymphopenia, artificial thymic organoids, ectrodactyly-ectodermal dysplasia clefting (EEC), ankyloblepharon-ectodermal defects-cleft lip/palate (AEC), cultured thymus tissue implantation (CTTI)

## Abstract

Pathogenic variants in the transcription factor *TP63* are associated with clinically overlapping syndromes including ectrodactyly-ectodermal dysplasia clefting (EEC) and ankyloblepharon-ectodermal defects-cleft lip/palate (AEC). T cell lymphopenia has rarely been described in individuals with *TP63* variants and the cause of the T cell defect is unclear. Here, we present a case of a female infant born with *TP63*-related syndrome and profound T cell lymphopenia, first uncovered through newborn screening. Flow cytometry analysis revealed low CD4+ naïve T cells and nearly absent CD8+ T cells with intact B and NK cell compartments. A *de novo* heterozygous pathogenic variant c.1040 G>A (C347Y) in exon 8 of *TP63* was identified. An artificial thymic organoid system, to assess the intrinsic ability of the patient’s hematopoietic cells to develop into T cells, was performed twice using separate peripheral blood samples. *Ex vivo* T cell differentiation was evident with the artificial organoid system, suggesting that a thymic stromal cell defect may be the cause of the T cell lymphopenia. Consistent with this, interrogation of publicly available data indicated that *TP63* expression in the human thymus is restricted to thymic epithelial cells. Based on these data, congenital athymia was suspected and the patient received an allogenic cultured thymus tissue implant (CTTI). This is the first report of suspected congenital athymia and attempted treatment with CTTI associated with *TP63* variant. At 9 months post-implant, peripheral lymphocyte analysis revealed measurable T cell receptor excision circles and presence of CD4+ recent thymic emigrants suggestive of early thymopoiesis. She will continue regular monitoring to ensure restoration of T cell immunity.

## 1 Introduction

Newborn screening for severe combined immunodeficiency (SCID) utilizes polymerase chain reaction to quantify T cell receptor excision circles (TRECs), a measure of normal T cell output from the thymus (1). SCID results from pathogenic variants in genes directly involved in the hematopoietic derived lymphocyte development and function (2). In addition to SCID, the TREC assay also identifies infants with hematopoietic extrinsic causes of T cell lymphopenia (TCL). Chromosome 22q11.2 deletion syndrome and pathogenic variants in *CHD7, TBX1, TBX2, FOXI3, FOXN1* and *PAX1* genes are known to be associated with thymic stromal cell defects (3–10). Recently, an *ex vivo* T cell differentiation assay using artificial thymic organoids (ATO) (11) has been described, which allows delineation of specific developmental blocks in T cell differentiation associated with SCID. This assay can help distinguish between hematopoietic intrinsic versus extrinsic causes of TCL (12, 13).

**Figure 1:**
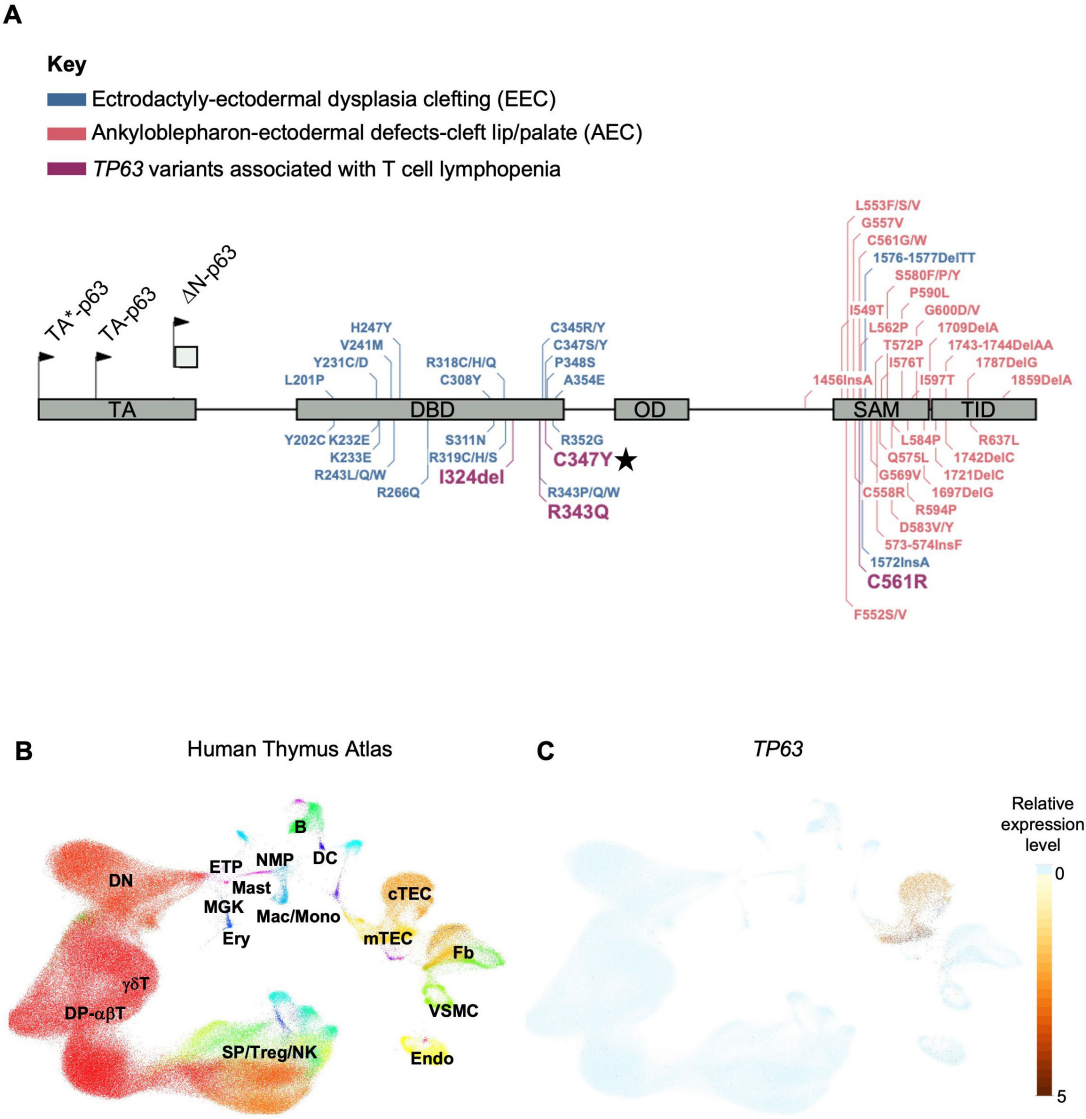
**(A)** Gene map of previously described *TP63* pathogenic variants and associated syndromes as indicated by color key. Given that there is overlap and variability in the features of AEC and EEC, variants have been categorized based on the most prevalent disease features that were reported. Numbering of pathogenic variants in TA*-p63 isoform is based on reference sequence NP_003713.3. Start codons for TA*-p63, TA-p63 and ΔN-p63 isoforms are indicated by arrows. TA Transactivation domain; DBD DNA binding domain; OD oligomerization domain; SAM sterile-α-motif domain; TID transcriptional inhibitory domain. Figure adapted from Osterburg et al. (16). The *TP63* variant identified in our patient is highlighted with a star. **(B)** Uniform manifold approximation and projection (UMAP) visualization showing the cellular composition of the human thymus obtained from the collection of single-cell datasets available on the UCSC Cell Browser [https://fetal-thymus.cells.ucsc.edu] (17). **(C)** Expression level of *TP63* gene in the human thymus atlas is restricted to cTEC and mTEC subsets. DN, CD4/CD8 double negative T cells; DP, CD4/CD8 double positive T cells; SP, CD4/CD8 single positive T cells; Treg, regulatory T cells; NK, natural killer cells; Endo, endothelial cells; VSMC, vascular smooth muscle cells; Fb, fetal bone; mTEC, medullary thymic epithelial cells; cTEC, cortical thymic epithelial cells; DC, dendritic cells; B, B cells; NMP, neutrophil myeloid progenitors; Mac/Mono, macrophages/monocytes; Mast, mast cells; MGK, megakaryocytes; ETP, early thymic progenitor; Ery, erythrocytes.

Pathogenic variants in *TP63*, a member of the p53-family of transcription factors, have been associated with TCL (**Table 1**). *TP63* encodes the transcription factor p63, which has several functions including epithelial development and progenitor cell regulation. *TP63* is comprised of five structural domains including the transactivation (TA) domain, DNA-binding domain (DBD), oligomerization domain (OD) (14), sterile-α-motif (SAM) domain and the transactivation inhibitory domain (TID) (15). Isoforms that either contain (TAp63) or lack (ΔNp63) the N-terminally located TA domain (**Figure 1A**) are differentially expressed in human tissues (18). ΔNp63 proteins are also expressed in cortical and medullary thymic epithelial cells (TECs) (**Figures 1B, C**) and in stratified epithelia where they play a central role in keratinocyte differentiation, proliferation, and cell adhesion (19). Mice that lack all isoforms of p63 do not develop limbs or stratified epithelia and have small, hypoplastic thymuses (20–22).

**Table 1:** Lymphocyte counts of individuals with *TP63* variant associated with T cell lymphopenia.

Individual	Variant (NM_003722.4)	*TP63* Domain	T cell count	Age (months)*					
				1	6	12	24	36	Reference
1	c.970_972delATT p.I324del	DBD	CD3+	**1136**	**835**	1932	2185		Giampietro et al., (36)
			CD4+	**829**	**605**	1449	1368		
			CD8+	**368**	**543**	527	721		
2	c.1028G>A, p.R343Q	DBD	CD3+	**1508**	**1536**	**1467**	1452	1433	Wenger et al., 2018 (37)
			CD4+	**1005**	**1092**	1079	940	955	
			CD8+	**461**	**404**	**304**	313	310	
3	c.1028G>A, p.R343Q	DBD	CD3+	**1313**	**1020**	1785	2208	1344	Wenger et al., 2018 (37)
			CD4+	**888**	**719**	1301	1500	881	
			CD8+	**386**	**278**	**363**	458	301	
4	c.1681T>C, p.C561R	SAM	CD3+	**1313**	**900**		**890**	1450	Helenius et al., (38)
			CD4+	**940**	**730**		550	700	
			CD8+	**180**	**120**		**140**	270	
5	c.1040 G>A, p.C347Y	DBD	CD3+	**108**	**158**	**179**	**191**		This report
			CD4+	**93**	**134**	**140**	**152**		
			CD8+	**ND**	**12**	**13**	**20**		

*Age based reference ranges: 1 month: CD3:2300-7000cells/µL; CD4: 1700-5300cells/µL; CD8: 400-1700cells/µL; 6 months: CD3:2400-6900cells/µL; CD4:1400-5100cells/µL; CD8:600-2200cells/µL;12 months: CD3:1600-6700cells/µL; CD4:1000-4600cells/µL; CD8:400-2100cells/µL; 24 months: CD3: 1400-8000cells/µL; CD4: 900-5500cells/µL; CD8:400-2300cells/µL; 36 months: CD3: 900-4500cells/µL; CD4:500-2400cells/µL; CD8: 300-1600cells/µL.

Numbers in bold indicate values below normal range.

*TP63* variants are associated with 6 overlapping clinical phenotypes - ectrodactyly-ectodermal dysplasia clefting (EEC), ankyloblepharon-ectodermal defects-cleft lip/palate (AEC), limb-mammary syndrome (LMS) and acro–dermato–ungual–lacrimal–tooth (ADULT) syndromes, as well as split-hand/foot malformation type 4 (SHFM4) and non-syndromic cleft lip (NSCL) (16, 23). Major clinical features of this group of disorders includes ectodermal dysplasia, cleft lip or palate, as well as hand and foot malformations (24). EEC is typically characterized by limb malformations including ectrodactyly, syndactyly, as well as thin, dry skin, nail dysplasia and sparse hair. AEC is characterized by more severe skin erosions, nail and dental abnormalities, in addition to ankyloblepharon (partial or complete adhesion of the upper and lower eyelid). Some genotype-phenotype correlation has been suggested for this group of disorders. Variants in the DBD are more frequently associated with EEC, whereas variants in the SAM domain and TID are more frequently associated with AEC (25). TCL is not commonly reported in the majority of described *TP63* variants. However, three out of four *TP63* variants associated with TCL localize to the DBD (**Figure 1A**).

Here we present an infant with overlapping features of EEC and AEC, as well as undetectable TRECs on newborn screen who was found to have a pathogenic *TP63* variant. With the use of the ATO co-culture system, we were able to determine that the patient’s TCL was likely due to a thymic, rather than hematopoietic, defect.

## 2 Case presentation

**Figure 2:**
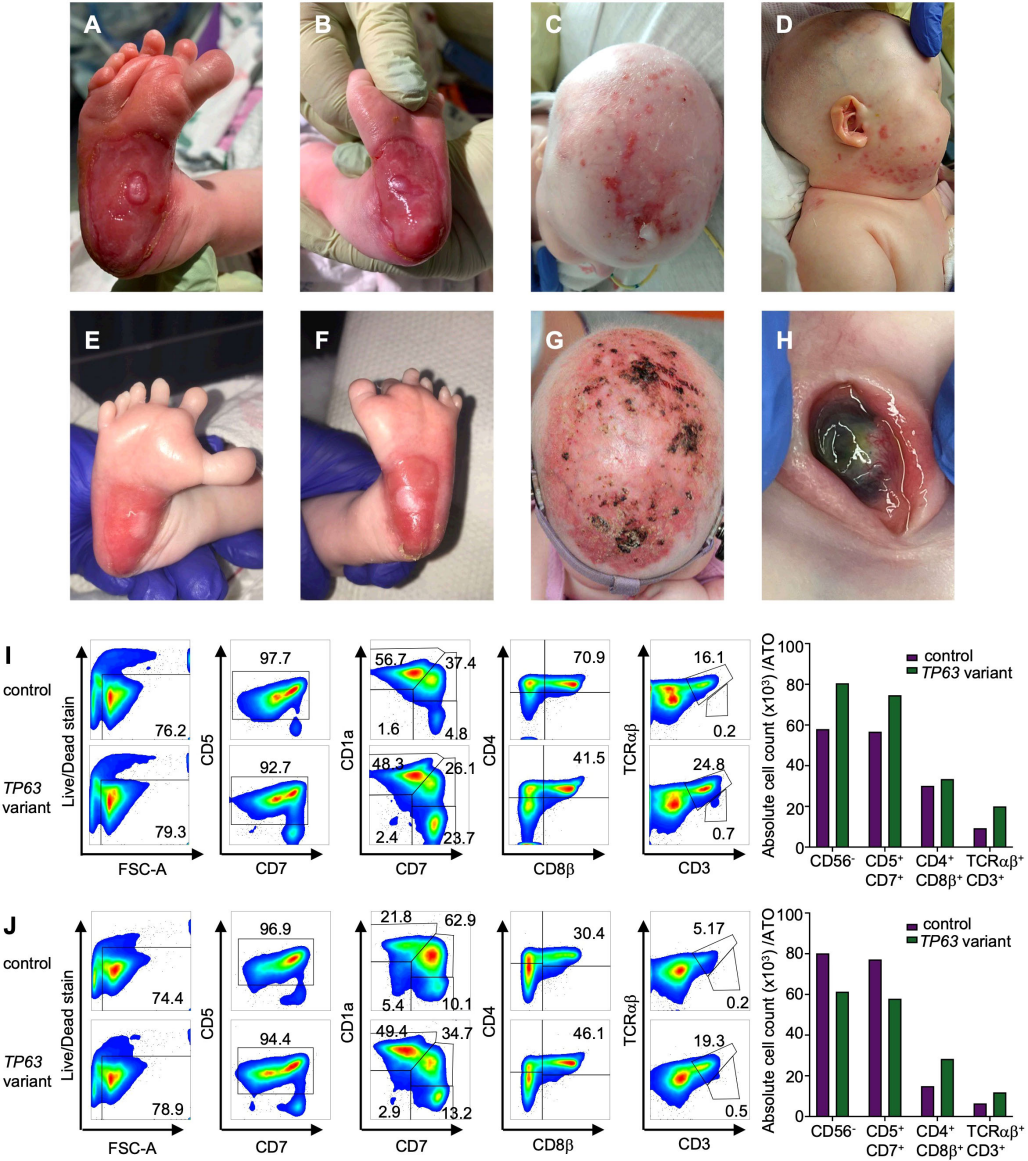
Clinical findings in a patient with *TP63* variant and T cell lymphopenia. **(A)** Right foot with widely spaced toes, polysyndactyly and severe skin erosion at the heel at 1 week of age **(B)** left foot with skin erosions at the heel at 1 week of age. **(C)** Severe skin erosions on scalp at 4 months of age **(D)** skin erosions on the right cheek at 4 months of age. **(E)** Right foot with improved skin erosions at 1 month of age **(F)** left foot with widely spaced toes, polysyndactyly and improved skin erosions at 1 month of age. **(G)** Scalp with improved skin erosions at 13 months of age **(H)** Left eye with central thinning at 13 months of age. **(I, J)** T cell differentiation induced by artificial thymic organoids culturing CD34^+^ cells from individual with *TP63* pathogenic variant (case) and unaffected age-matched control in the Artificial Thymic Organoid (ATO) system. Representative analysis of T cell differentiation in case and control at 4 months of age **(I)** and 26 months of age **(J)** after 6 weeks in culture in the ATO system. Cells were gated on LIVE/DEAD^–^CD45^+^CD14^–^CD56– cells and the expression of early and late T cell commitment markers (CD5, CD7, CD1α, CD4, CD8β, CD3, TCRαβ. Absolute cell counts per artificial thymic organoid and indicated early and late T cell commitment markers in *TP63* variant (purple) and control (green) at 4 months **(I)** and 26 months of age **(J)**.

The patient is a 3-year-old girl born at term to healthy, non-consanguineous parents. No pertinent family history was identified. She required continuous positive airway pressure, then intubation, at birth due to micrognathia and tongue-base airway obstruction. Multiple congenital anomalies were noted including features of AEC, such as bilateral blepharophimosis, cloudy corneas, diffuse areas of denuded skin, particularly on her scalp and feet and cleft palate. In addition to her cleft palate, features consistent with EEC included complex hand anomalies (i.e. anterior polydactyly; 3, 4 syndactyly; unilateral ectrodactyly), hypoplastic nails and foot anomalies (i.e. bilateral ectrodactyly, posterior polydactyly, hypoplastic nails) (**Figures 2A–H**). She was also noted to have an atrial septal defect (ASD), imperforate anus and rectovestibular fistula. Heart defects and anorectal malformations have rarely been reported in individuals with *TP63* variants (26, 27). Newborn screens were notable for 0 TREC copy number/μL of blood at 14 hours, 7, 19 and 30 days of life (**Table 2**). Lymphocyte subsets at 2 weeks of life revealed low CD3+ cells (30 cells/μl), low CD4+ cells (30 cells/μl), absent CD8+ cells, normal CD19+ (1029 cells/μl), CD16+ (418 cells/μl) and CD56+ (239 cells/μl), which was consistent with a T-B+NK+ severe combined immunodeficiency (SCID)-like phenotype (**Table 2**).

**Table 2:** Patient immune studies over time.

	Age (months)					Months post-cultured thymus tissue implantation*				Reference range
	0	3	9	18	24	0	3	6	9	
T cells										
CD3+	30 (2%)	128 (8%)	145 (14%)	200 (18%)	191 (29%)	0 (0.1%)	21 (9%)	58 (19%)	200 (42%)	1400-8000 cells/μl (39-73%)
CD4+	30 (2%)	96 (6%)	114 (11%)	145 (13%)	152 (23%)	0 (0%)	18 (8%)	34 (11%)	119 (25%)	900-5500 cells/μl (25-50%)
CD8+	- (<1%)	- (<1%)	-(<1%)	22 (2%)	20 (3%)	0 (0%)	0 (<1%)	9 (3%)	33 (7%)	400-2300 cells/μl (11-32%)
CD4+ CD45RA+	12%	4%	2%	5%	4%	0%	<1%	7%	14%	63-91%
CD4+ CD45RO+	70%	80%	79%	84%	69%	0%	90%	84%	79%	7- 20%
CD8+ CD45RA+	69%	21%	18%	24%	21%	0%	2%	4%	8%	71- 98%
CD8+ CD45RO+	2%	38%	33%	48%	39%	0%	60%	69%	67%	2- 12%
TCRαβ		85%	90%	80%	86%	0%	90%	58%	66%	90- 99%
TCRγδ		15%	10%	20%	14%	0%	10%	42%	34%	1-9%
TREC^†^	0			BLD	BLD		BLD	BLD	570	>6794 copies/μl
CD4+ RTE				11.3 (6.8%)	6.6 (4.1%)		0.2 (1.1%)	1.9 (5.1%)	19.8 (14.6%)	170.0−1007 cells/ μl (26-68%)
B cells										
CD19+	1029 (69%)	1089 (68%)	703 (68%)	645 (58%)	349 (52%)	185 (64%)	120 (52%)	150 (49%)	209 (44%)	600-3100 cells/ μl (17-41%)
IgG	497	465	828^††^	671^††^	928^††^		996^††^	1080^††^	887^††^	468-1196 mg/dL
IgM	48	58	52						54	47 - 200 mg/dL
IgA	<7	15	20						43	21 - 117 mg/dL
NK cells										
CD16+	418 (28%)	352 (22%)	145 (14%)	234 (21%)	86 (37%)	60 (21%)	85 (37%)	86 (28%)	62 (13%)	45-1101 cells/μl (4-22%)
CD56+	239 (16%)	272 (17%)	134 (13%)	200 (18%)	79 (29%)	61 (21%)	67 (29%)	71 (23%)	52 (11%)	0-1206 cells/ μl (2-24%)
Mitogen stimulation										
PHA-induced lymphocyte proliferation	5.0%		56.8%		9.7%					% of control response

^*^Patient underwent cultured thymus tissue implantation at 28 months of age.

†quantification of TRECs from newborn screen at 14 hours and 7 days of life by qPCR and subsequent TREC analysis by RT qPCR at 18, 24, 31, 34 and 37 months of life.

††values obtained while patient was receiving human immune globulin.

BLD, below level of detection.

Initial testing demonstrated normal adenosine deaminase (ADA) and purine nucleoside phosphorylase (PNP) enzymes and normal double-strand DNA break repair in lymphocytes 24 hours post-irradiation. No maternal engraftment was detected. Genetic studies included microarray analysis, which revealed three copy number variations at 1q44 (36kb copy gain), 3q26.1 (74kb copy gain) and 18p11.32 (66kb copy loss). However, all three represent population variants that are likely nonpathogenic. She underwent rapid whole genome sequencing through the SeqFirst research program at the University of Washington (IRB # STUDY00008810), which revealed a heterozygous *de novo* variant in exon 8 of *TP63* corresponding to c.1040 G>A with predicted protein change C347Y in the DBD of p63 (**Figure 1**). This variant has been previously reported in patients with syndromic features consistent with AEC and with EEC syndrome (25, 28, 29). An additional heterozygous variant of uncertain significance (VUS) was identified in *PRKDC* (c.6901C>G; Q2301E), which was also present in the patient’s father who was healthy. Given that mutations in *PRKDC* have been associated with immunodeficiency (30–32), targeted RNA sequencing to assess for altered splicing or expression levels was performed. No significant changes in *PRKDC* transcript levels or splicing patterns were detected (data not shown). Additionally, the patient’s normal DNA repair studies and normal B cell counts further argued against the contribution of PRKDC associated immunodeficiency.

To determine whether the cause of the patient’s T cell lymphopenia was due to a thymic or hematopoietic defect, we took advantage of the recently developed artificial thymic organoid (ATO) system (11, 12). Upon parental informed consent, the patient was enrolled in protocol NCT03610802 approved by the National Institute of Health’s Institutional Review Board. CD34+CD3- cells were isolated from the patient’s peripheral blood mononuclear cells (PBMCs) and cultured in the ATO system. T cell maturation was analyzed after 6 weeks in culture in the ATO system both at 4 and at 26 months of age. **Figures 2I, J** show the flow cytometry gating strategy and absolute T cell counts of cells at different stages of intrathymic development. We found equivalent numbers of total cells (CD56-CD14-), T cell lineage committed cells (CD5+CD7+), double positive (DP) cells (CD4+CD8β+) and mature CD3+TCRαβ+ cells in the patient as compared to an unaffected control. These data suggested that the patient’s TCL was extrinsic to the hematopoietic cell lineage and most likely due to a thymic stromal cell defect in the setting of her *TP63* variant. Consistent with this, interrogation of a publicly available database of gene expression profile in the human thymus demonstrated that *TP63* expression is restricted to cortical and medullary thymic epithelial cells, and is not observed in thymic hematopoietic cell types (**Figures 1B, C**) (17).

Over time, the patient remained with persistently low numbers of T cells, high percentage of TCRγδ+ cells and low absolute counts of TCRαβ+ cells (**Table 2**). TRECs assessed at 18 and 24 months of age remained below the level of assay detection (**Table 2**). Within the T cell subset, cells displayed a predominantly memory CD4+CD45RO+ phenotype. The patient was started on IgG replacement at 4 weeks of life; thus, IgG levels reflect IgG replacement. However, she had normal levels of IgM, as well as normal levels of IgA and IgE suggesting adequate class-switching, presumably through B-cell interaction with γδT cells (33). Mitogen-induced lymphocyte proliferation was assessed at 2 weeks, 9, and 21 months of age and demonstrated a reduced response to phytohemagglutinin (PHA) stimulation when compared to control lymphocytes, which may reflect the patient’s low lymphocyte count (34). Recent thymic emigrants were reported as 11.3 CD4+ cells/μl (5.6%) and 6.6 CD4+ cells/μl (4.4%) [reference range 170-1007 cells/μl (25.8-68%)] suggesting no recovery of T cell counts by 18 and 24 months of age, respectively.

The patient’s clinical course (**Supplementary Figure 1**) was characterized by extubation to high flow nasal cannula by 1 month of age and subsequent weaning to room air. She had severe obstructive sleep apnea resulting in the need for supplemental oxygen during sleep. Due to her dysphagia and faltering growth, she required post-pyloric feeds, followed by gastrostomy tube feeds at 4 months of age. In the setting of her anorectal malformation, she initially had a colostomy placed and then underwent an anorectoplasty at 7 months of age. Eye exams demonstrated neovascularization and thinning of bilateral corneas (**Figure 2H**), which led to corneal perforation of both eyes requiring Gundersen conjunctival flap repair and corneal transplant. She has hearing impairment requiring hearing aids, as well as chronic otorrhea. Her cleft palate was closed at 16 months of age. Echocardiogram demonstrated normal systolic function and spontaneous closure of ASD by 18 months of age. She requires daily emollient application to her skin, as well as antibacterial and antifungal topical ointments. Given her TCL, she receives immunoglobulin replacement therapy, trimethoprim/sulfamethoxazole, fluconazole, azithromycin, and acyclovir prophylaxis. She has been kept isolated and has not had significant infectious complications.

In light of her persistent TCL and suspected athymia, she underwent cultured thymic tissue implantation (CTTI) at 28 months of age. Because her total CD3+ T cell counts were > 100 cells/µl and she had normal proliferation responses to PHA mitogen prior to implantation, she received immunosuppression with anti-thymocyte globulin and cyclosporin to reduce the risk of allograft rejection (35). She has been maintained on cyclosporin following implant without complications. At 6 months post-CTTI no significant improvement in thymic output was appreciated. Encouragingly, at 9 months post-CTTI evidence of measurable TRECs and CD4+ recent thymic emigrants (RTE) were noted, along with a 25% increase in total circulating CD4+ T cells (**Table 2**) suggesting early thymopoiesis. Thymopoiesis typically occurs between 6 and 12 months post-CTTI (10, 35). She will continue regular monitoring to ensure thymic recovery continues and circulating T cells are functional.

## 3 Discussion

Our patient’s case adds to a growing body of literature suggesting that individuals with *TP63* variants can present with T cell lymphopenia. Four such individuals have been described previously, including a set of monozygotic twins (36–38). As in our patient, the other four individuals were identified on newborn screen as having low or absent TRECs. However, in all the published reports, the T cell lymphopenia was not severe and resolved by 1-3 years of age (**Table 1**). The individual described by Giampietro et al. initially demonstrated mild T cell lymphopenia, but intact lymphocyte response to PHA stimulation. His course was complicated by *Pseudomonas aeruginosa* periorbital cellulitis at 2 months of age, and he remained on intravenous immune globulin replacement (IVIG) and did not receive live vaccines until his lymphocyte counts spontaneously recovered at 14 months of age (36). Two monozygotic twins reported by Wenger et al. had low overall CD3, CD4 and CD8 counts that recovered by 12 months of age. Their lymphocytes also responded appropriately to PHA stimulation. Around the time of T cell recovery, both twins demonstrated normal effector and memory CD4+ and CD8+ T cell distribution. Both twins had mildly low IgG and IgM levels in infancy that recovered, and this was thought to be due to transient hypogammaglobulinemia of infancy. They did not require IVIG, and tolerated live vaccines once their T lymphocyte counts normalized. At 19 months of age, Twin 1 was hospitalized with a skin abscess caused by Methicillin Resistant *Staphylococcus aureus* (MRSA) complicated by toxin-mediated Staphylococcal Scalded Skin Syndrome. Twin 2 had MRSA orbital cellulitis around the same time, but did not require hospitalization. In addition, Twin 2 had *E. coli* pyelonephritis in infancy and was found to have vesicoureteral reflux (37). Helenius et al. described a preterm infant with overlapping features of EEC/AEC and lymphopenia. Interestingly, while all of the other known individuals with T cell lymphopenia have a *TP63* variant in the DNA-binding domain, this individual’s *TP63* variant is in the SAM domain (**Figure 1**). She had low CD3, CD4, and CD8 counts that recovered by age 3. She initially had undetectable IgA/IgM and low IgG and was started on IVIG and trimethoprim/sulfamethoxazole prophylaxis in the neonatal period and continued through age 30 months. She developed Staphylococcal sepsis on four separate occasions in the first four months of life, but then continued without additional serious bacterial infections (38). Finally, two years before the genetic cause of EEC and AEC was identified (39), Frick et al. reported a newborn with an undefined genetic variant resulting in EEC who was born at 35 weeks due to maternal eclampsia, and who died shortly after birth. Post-mortem examination revealed a small rudimentary thymus and lack of Hassall’s corpuscles (40). Compared to previously published cases, our patient demonstrated severe and persistent T cell lymphopenia (**Table 1**), prompting intervention with CTTI. To our knowledge, she is the first patient with *TP63* variant and suspected congenital athymia in whom definitive treatment with cultured thymic tissue implantation has been attempted.

Variants in the DBD of *TP63* have been more frequently associated with EEC. Our patient highlights the clinical variability and phenotypic overlap among *TP63*-related disorders. The presence of hand and foot malformations, as well as imperforate anus aligns with features found in EEC. However, the severity of her skin erosions and the presence of bilateral blepharophimosis is consistent with features of AEC. Interestingly, another infant with the same *TP63* variant (c.1040G > A, p.C347Y) was also reported to have overlapping features of EEC and AEC. Unfortunately, she died at 2 months of age due to sepsis and it is unknown whether she had TCL (28). *TP63* variant c.1039T>A, p.C347S has been reported in an individual with features of EEC and recurrent pulmonary infections (29). With the wide adoption of the TREC assay in NBS, more individuals with *TP63* variants and TCL will likely be identified in the neonatal period when initiation of immunoglobulin replacement therapy and antimicrobial prophylaxis can prevent infectious complications.

It remains unclear why some individuals with *TP63* variants develop T cell lymphopenia and why it is more severe and persistent in some and spontaneously resolves in others. In mouse models, the T cell lymphopenia appears to be linked to the loss of the ΔNp63. The *p63*-deficiency blocks the proliferation of thymic epithelial stem cells prenatally (21, 22). In the postnatal period, a recent study using conditional knockout of p63 in TECs showed that mice develop profound thymic hypoplasia, lack normal thymic architecture and have T cell differentiation defects (41). Based on these data, it is possible that TCL seen in individuals with *TP63* variants is due to a direct effect of those variants on development and function of TECs. Furthermore, as demonstrated in *FOXN1* variants, the variability in clinical phenotype in *TP63* variants may be determined based on how the variant impacts transcriptional activity, nuclear localization and/or dominant negative effects (5). Indeed, two recent studies shed light on the mechanism of specific variants on transcriptional activity of *TP63*. Russo et al. show that several *TP63* variants associated with AEC and that localize to the SAM domain cause decreased DNA binding and transcriptional activity due to TP63 protein aggregation (23). In a separate study, this group performed a comprehensive analysis of variants underlying EEC on DNA binding. Interestingly, they show that the C347Y variant found in our patient maps to a *TP63* region responsible for direct DNA contact and does not bind DNA as demonstrated in their DNA pull down, p53 displacement and surface plasmon resonance assays evaluating protein-DNA interaction. Thus, the C347Y substitution is very damaging. In contrast, the C343S variant demonstrated wildtype-like DNA binding. Similarly, R343Q variant, identified in the monozygotic twins (**Table 1**), localizes to the TP63 protein interface with direct DNA contact and shows absent transcriptional activity and reduced Zinc binding (16). More studies are needed to understand why variants that similarly impact DNA binding to TP63 result in such heterogenous clinical phenotypes and varying impact on T cell development.

With regard to persistence of TCL, homeostatic expansion of T cells in the periphery may explain the spontaneous resolution of TCL in some patients. It is possible that this could lead to underreporting of TCL in patients with *TP63* variants. Individuals with thymic defects due to 22q11.2 deletion syndrome and *FOXN1* haploinsufficiency can have a marked T cell lymphopenia early in life with a gradual normalization of CD4+ and CD8+ T cell numbers possibly due to homeostatic expansion (42, 43). It has been proposed that in the absence of normal thymic development, homeostatic proliferation may result in a restricted T cell receptor repertoire and, subsequently, suboptimal T cell responses in the setting of infection or expansion of cells that react to self-antigen, leading to autoimmunity (44).

Infants with TCL who are identified on NBS can have a hematopoietic or non-hematopoietic cause of their lymphopenia and choosing the correct and timely therapy can be clinically challenging. With the wide adaptation of SCID NBS, an increasing number of patients are being recognized as having thymic defects or genetically undefined causes of SCID. Early recognition and prompt referral for CTTI leads to better clinical outcomes (45). Our case highlights the difficulty of making therapeutic decisions for treating SCID when a genetic variant is identified, but there is very little known about the effect of that gene variant on immune system functioning. The ATO assay demonstrated that the patient’s T cell precursors were capable of fully differentiating ***ex vivo*** into single positive CD4 and CD8 T cells suggesting that CTTI may provide definitive treatment for her T cell lymphopenia. The ATO co-culture system also has some limitations. For example, some metabolic causes of T cell lymphopenia, such as in adenosine deaminase deficiency, are not accurately captured by the assay resulting in T cell differentiation *ex vivo* (46). Our case also highlights the time needed for additional investigation, as well as a period of observation to determine if the TCL may resolve without intervention, as was seen with other individuals with *TP63* variants. Nevertheless, the ATO co-culture system is a promising diagnostic tool that can help identify infants with thymic defects and novel genetic causes of SCID (46).

## Data Availability

The original contributions presented in the study are included in the article/**Supplementary Material**, further inquiries can be directed to the corresponding author/s.

## Supplementary material

Supplementary Figure 1 Visual representation of the clinical time course and treatment of patient with *TP63* variant and T cell lymphopenia. NBS, newborn screen; GT, Gastrostomy tube; OSA, obstructive sleep apnea; PSARP, Posterior Sagittal Anorectoplasty; ASD, atrial septal defect; ATG, anti-thymocyte globulin.
