# Supplementary material for: Case report: Artificial thymic organoids facilitate clinical decisions for a patient with a TP63 variant and severe persistent T cell lymphopenia
Source: Front Immunol. 2024 Sep 18;15:1438383. doi: 10.3389/fimmu.2024.1438383 (PMC11448704; doi:10.3389/fimmu.2024.1438383)
Supplement: Supplementary Figure 1 — Visual representation of the clinical time course and treatment of patient with TP63 variant and T cell lymphopenia. NBS, newborn screen; GT, Gastrostomy tube; OSA, obstructive sleep apnea; PSARP, Posterior Sagittal Anorectoplasty; ASD, atrial septal defect; ATG, anti-thymocyte globulin. [file DataSheet1.pdf]

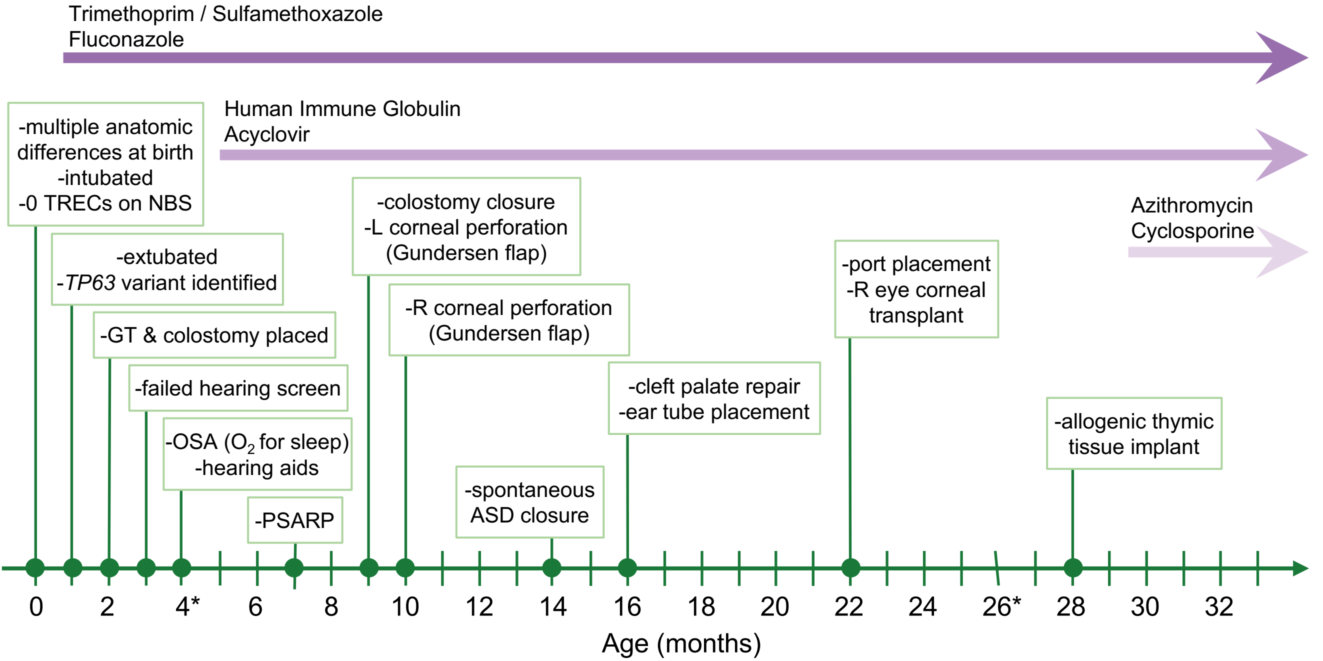
Trimethoprim / Sulfamethoxazole
Fluconazole
Human Immune Globulin
Acyclovir
Azithromycin
Cyclosporine
-multiple anatomic differences at birth
-intubated
-0 TRECs on NBS
-extubated
-*TP63* variant identified
-GT & colostomy placed
-failed hearing screen
-OSA (O_2 for sleep)
-hearing aids
-PSARP
-colostomy closure
-L corneal perforation (Gundersen flap)
-R corneal perforation (Gundersen flap)
-spontaneous ASD closure
-cleft palate repair
-ear tube placement
-port placement
-R eye corneal transplant
-allogenic thymic tissue implant
0
2
4*
6
8
10
12
14
16
18
20
22
24
26*
28
30
32
Age (months)
